# Aggressiveness-guided nodule management for lung cancer screening in Europe—justification for follow-up intervals and definition of growth

**DOI:** 10.1007/s00330-025-11647-5

**Published:** 2025-07-01

**Authors:** Mathias Prokop, Cornelia Schaefer-Prokop, Colin Jacobs, Annemiek Snoeckx, Jürgen Biederer, Thomas Frauenfelder, Fergus Gleeson, Hans-Ulrich Kauczor, Anagha P. Parkar, Rozemarijn Vliegenthart, Marie-Pierre Revel, Mario Silva, Helmut Prosch

**Affiliations:** 1 Department of Medical Imaging, Radboud University Center, Nijmegen, The Netherlands; 2 Department of Radiology, University Medical Center Groningen, Groningen, The Netherlands; 3 Department of Radiology, Meander Medical Center, Amersfoort, The Netherlands; 4 Department of Radiology, Antwerp University Hospital, Edegem, Belgium; 5 Faculty of Medicine and Health Sciences, University of Antwerp, Wilrijk, Belgium; 6 Department of Biomedical Imaging and Image-Guided Therapy, Medical University of Vienna, Vienna General Hospital, Vienna, Austria; 7 Department of Diagnostic and Interventional Radiology, University Hospital of Heidelberg, Heidelberg, Germany; 8 Translational Lung Research Center Heidelberg (TLRC), Member of the German Lung Research Center (DZL), Heidelberg, Germany; 9 University of Latvia, Faculty of Medicine, Riga, Latvia; 10 Institute of Diagnostic and Interventional Radiology, University Hospital Zurich, University of Zurich, Zurich, Switzerland; 11 Department of Oncology, University of Oxford, Oxford, UK; 12 Department of Radiology, Haraldsplass Deaconess Hospital, Bergen, Norway; 13 Department of Clinical Medicine, Faculty of Medicine and Dentistry, University of Bergen, Bergen, Norway; 14 Department of Radiology, University Medical Center Groningen, University of Groningen, Groningen, The Netherlands; 15 Department of Radiology, Cochin Hospital, Université Paris Cité, Paris, France; 16 Scienze Radiologiche, Department of Medicine and Surgery (DiMeC), University of Parma, Parma, Italy

**Keywords:** Lung cancer, Pulmonary nodule, Screening programs (Diagnostic), Artificial intelligence, Low-dose computed tomography

## Abstract

**Abstract:**

The European Society of Thoracic Imaging (ESTI) nodule management recommendation for lung cancer screening with low-dose CT builds on existing nodule management guidelines but puts a stronger focus on lesion aggressiveness and measurement error. Key objectives included finding a compromise between the overall number of follow-up examinations, avoiding a major stage shift, and reducing the risk for overtreatment. Nodule management categories at baseline are chosen depending on the size of a solid nodule or the solid component of a subsolid or cystic nodule, with suspicious morphology upgrading risk to the next higher category. Higher risk categories mandate shorter follow-up times or diagnostic workup. Volume is the preferred size measure, with diameter measurements as a fallback if segmentation for volumetry is inaccurate at visual control. Nodule aggressiveness at follow-up is estimated from growth rate, calculated as volume doubling time (VDT), or yearly diameter change. Calculation of growth rate, however, is strongly affected by measurement variability, with large error margins for short follow-up and slower growing lesions. Growth thresholds were therefore set so that rapidly growing lesions can be identified while still small, while unnecessary workups for benign or slow-growing lesions could be kept low. New lesions that are retrospectively visible on earlier scans are managed according to their growth rate. New nodules not visible on earlier scans are followed after 3 months if they have a volume of ≥ 30 mm^3^.

**Key Points:**

***Question*** *This work strives to reduce follow-up examinations while preventing major stage shift and overtreatment. It provides nodule management based on estimated nodule aggressiveness*.

***Findings*** *Calculation of the growth rate of pulmonary nodules is strongly affected by measurement variability, with large error margins for short follow-up and slower growing lesions*.

***Clinical relevance*** *Growth thresholds that trigger management are adjusted to the follow-up time so that rapidly growing lesions can be identified while still being small while unnecessary workups for benign or slow-growing lesions can be reduced*.

The European Society of Thoracic Imaging (ESTI) nodule management recommendation for lung cancer screening with low-dose computed tomography (CT) (LDCT) (Figs. 1 and 2 and Table 1) [1] builds on existing nodule management guidelines but puts a stronger focus on estimated lesion aggressiveness and measurement error. Key objectives were finding a compromise between the overall number of follow-up examinations, avoiding major stage shift, and reducing the risk of overtreatment. The rationale for the choices made is discussed below. This nodule management recommendation does not cover incidentally detected nodules on clinical scans.

**Fig. 1:**
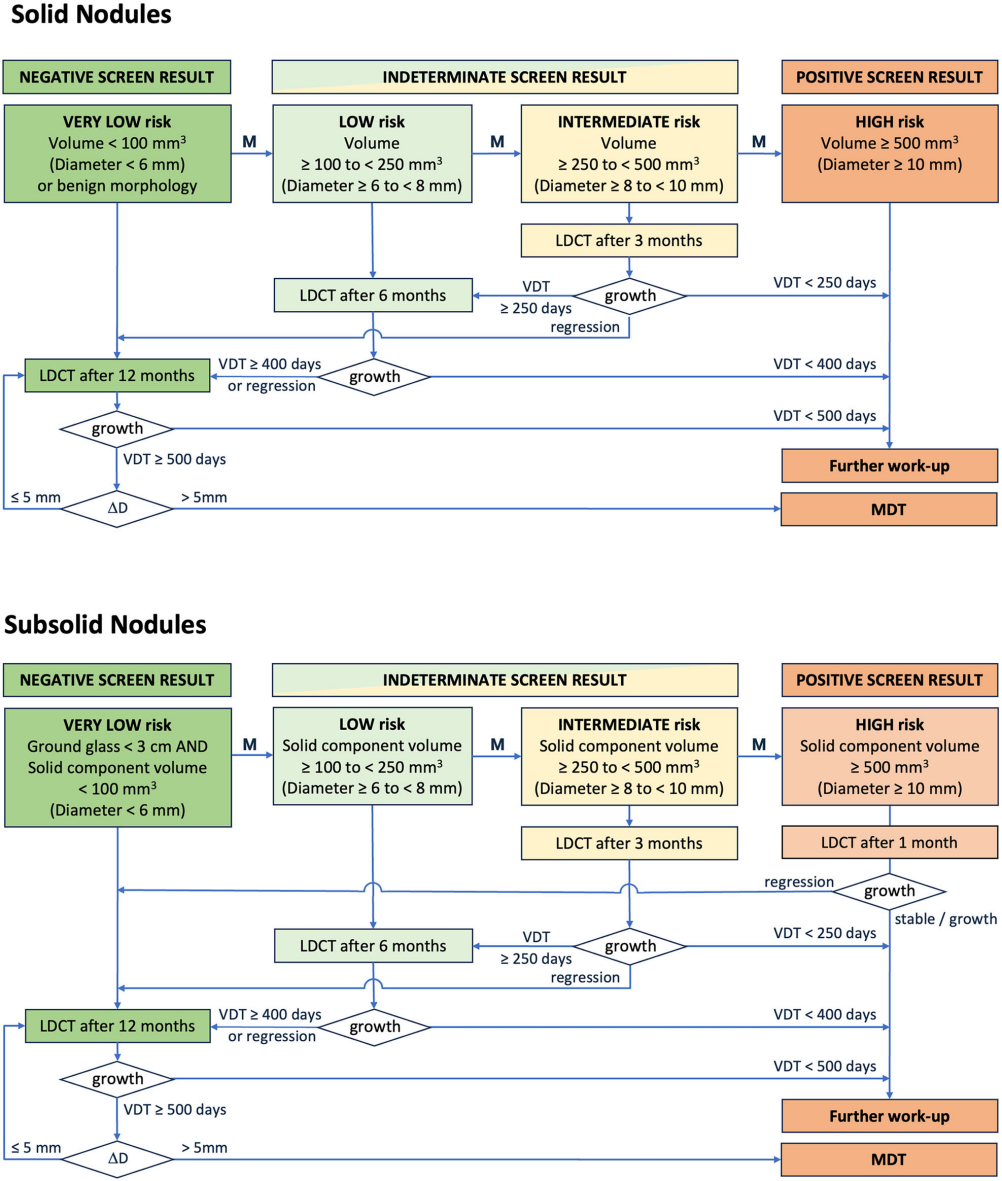
Flowchart for management of solid and subsolid nodules detected at baseline. M *= *suspicious morphology upgrades risk to next category: spiculation, architectural distortion (pleural tag, fissure displacement), cystic component, bubble-like lucencies, concave sign, and narrowed vessels. Benign morphology: calcification (central, diffuse, 7 popcorn-like), fat components, typical intrapulmonary lymph node morphology (smooth margins, oval, lentiform or triangular shape, < 1 cm, distance to pleura < 1 cm, under the carina). Growth *= *substantial growth, defined as follows: If volumetry is possible: VDT < 250 days at 3 months, VDT < 400 days at 6 months, and VDT < 500 days at ≥ 12 months. If volumetry fails: visually verifiable increase in average diameter of > 1.5 mm over a time interval of maximally 1 year, or substantial change in morphology. A decrease in size may indicate a benign process (inflammation, infection, other) and prompts ongoing follow-up to ensure shrinkage continues. Solid component: if the solid component of a part-solid nodule is more than 80% of the entire nodule diameter, this nodule should be classified as a solid nodule. Δ*D* *= *change in effective diameter relative to baseline, derived from volume or from manual measurements if volumetry fails. MDT = multidisciplinary team decision is advised if the effective diameter of a slow-growing nodule increases by more than 5 mm from baseline.

**Fig. 2:**
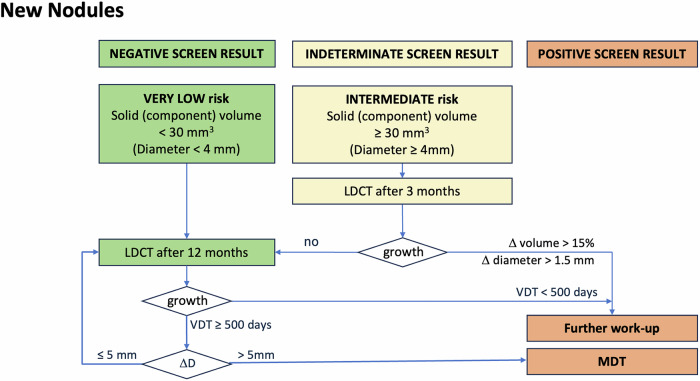
Flowchart for management of new nodules. New nodules that had been missed or not reported on previous scans are managed according to the same rules at nodules found at baseline

**Table 1:** Estimated growth speed, indicated as VDT, for new nodules presenting at follow-up

		Slowest VDT if size at current scan =			
Follow-up interval	Initial size	30 mm^3^	100 mm^3^	250 mm^3^	500 mm^3^
3 months	4 mm^3^	31 d	20 d	15 d	13 d
6 months	4 mm^3^	63 d	39 d	31 d	26 d
12 months	4 mm^3^	126 d	79 d	61 d	52 d
3 months	15 mm^3^	91 d	33 d	22 d	18 d
6 months	15 mm^3^	183 d	67 d	45 d	36 d
12 months	15 mm^3^	365 d	133 d	90 d	72 d

We assumed that the nodule was present at the earlier scan, but below a certain initial size limit that precluded detection on the earlier scan. Depending on this detection limit, the VDT needed to reach the size at detection varies substantially. For most scanners, a detection limit of 4 mm^3^ (2 mm effective diameter) is a conservative estimate, but for NELSON, a limit of 15 mm^3^ (3 mm effective diameter) was chosen [19]

## Design principles

Regular lung cancer screening is performed at one-year intervals. Nodule management is explicitly not only based on the risk for malignancy but, where possible, based on the estimated aggressiveness of the nodule, to avoid overdiagnosis and overtreatment of the least aggressive lesions. The nodule with the shortest follow-up interval determines participants’ management. Clearly benign nodules do not affect management.

Nodules are categorised into four categories (very low, low, intermediate, and high) that indicate their risk of major stage shift (developing into a tumour stage of T1c or higher) within 1 year. Management is defined by category. Lack of substantial growth at follow-up will downgrade a nodule to the next lower category. Decrease in size, with total or partial regression, suggests a benign process (e.g., inflammation, infection, others) and will downgrade a nodule to the lowest risk category and regular 1-year follow-up.

### Baseline scans

At baseline screening, growth information is not yet available for assessing the risk of malignancy and for estimating the aggressiveness of a lesion. Aggressiveness is mainly derived from nodule type: solid, part-solid, or non-solid (pure ground-glass). The risk of malignancy is further related to nodule size, morphology, location, demographic information, such as age and sex, and non-nodule related information on CT images, such as the presence of emphysema or interstitial abnormalities [2, 3].

We chose to only use nodule type, size, and suspicious morphology for nodule management at baseline. Suspicious morphology provides the option to upgrade nodule risk by one risk category (Fig. 1). In the future, management at baseline may be supported by automatic assignment of risk categories by artificial intelligence (AI) [4, 5]. However, prospective studies are needed that investigate how these AI-based risk scores can be integrated into nodule management.

### Follow-up

The ESTI nodule management recommendation explicitly acknowledges measurement variability as a factor that needs to be considered when defining growth thresholds and follow-up intervals. It is designed to significantly reduce the likelihood of a major stage shift during follow-up, defined as a T1a tumour becoming a tumour of stage T1c or higher. Stage shift from T1a (< 1 cm diameter) to T1b cannot be avoided because some lesions will exceed the size of 1 cm at follow-up. However, outcomes quickly deteriorate with higher T-stages, which is why we aim to avoid tumours growing to T1c or T2, stages, in which the risk for lymph node metastases grows. The purpose of the first follow-up is to identify fast-growing malignant nodules; subsequent follow-ups are designed to identify slower-growing nodules.

Growth thresholds are chosen so that only very few diagnostic workups for benign nodules are expected. From the second follow-up onward, when growth rate has been established, the ESTI nodule management recommendation is designed to not only avoid major stage shift but also to keep diameter growth below 5 mm for the vast majority of all followed nodules.

## Growth and measurement errors

### Size measurement

Nodule size measurements using calipers are substantially less accurate and reproducible than volumetric measurements [6, 7]. However, nodule volumes are less intuitive to interpret than diameters, which is why we translate volumes, where necessary, back to effective diameters using the diameter of a sphere of the same volume. This also allows for better comparison with other guidelines and the TNM staging system, even though T-staging uses the largest diameter instead of the effective diameter.

In this nodule management recommendation, we use nodule volumetry as the primary way to assess nodule size. Only if volumetry fails, do manual diameter measurements have to be performed. Importantly, the same evaluation tool needs to be used across follow-up scans, be it volumetry or manual measurement.

### Nodule volumetry

Nodule size should therefore be measured by volumetry, which involves software that performs nodule segmentation and calculates the volume of the segmented nodule. While all available software programs will segment solid nodules, more advanced versions are necessary for segmenting subsolid nodules, with separate measurements for the total nodule and potential solid components. For follow-up, the same segmentation software should be used to improve the reproducibility of measurements.

If participants are followed at a different centre from that in which the first interpretation was performed, or if there was a major update or change in the volumetry software used, then the previous exam should be re-evaluated with the new volumetry software in order to calculate growth.

If the accuracy of fully automated segmentation is not acceptable, adjustment of segmentation parameters is encouraged for a better segmentation result. Manual adjustment of segmentation in regions of over- or under-segmentation is allowed. Since the difference in reproducibility between manual diameter measurements and volumetry is so large, even a non-optimum but acceptable volumetry [7, 8] is better than manual diameter measurements. Not all volumetry software, however, allows for these adjustments.

Segmentation failure that cannot be corrected triggers manual diameter measurements. Failure of volumetry is based on the definition of segmentation accuracy introduced by De Hoop et al [7]. For this management recommendation, we define failure of volumetry as category “(3) ‘Poor’: part of the nodule is segmented, but the segmented volume is not representative of the nodule (estimated mismatch > 20%)” or category “(4) ‘Failure’: no segmentation or the result has no similarity with the lesion.”

### Manual diameter measurements

Average and effective diameters do not provide the same numbers; the average diameter will provide a slightly larger value than the effective diameter obtained by volumetry, especially in irregularly shaped lesions. We therefore used average diameter thresholds that were rounded up to slightly higher numbers than the corresponding effective diameters derived from volumetry (6 mm instead of 5.8 mm; 8 mm instead of 7.8 mm; and 10 mm instead of 9.8 mm).

If adjustments of volumetry are not possible or feasible in case of failed segmentation, evaluation must resort to manual diameter measurements. We expect this to mostly happen with attached nodules, cystic nodules and measurements of the solid core of subsolid lesions, which are difficult to tackle by automated volumetry. Manual measurements should be performed as the largest orthogonal dimensions in axial sections and in the craniocaudal direction. Manual measurements should be given to one decimal point in mm. The three measurements are then averaged.

If volumetry software is updated and becomes good enough to segment a lesion during follow-up, all previous measurements may be repeated by volumetry to assess growth. If this is not possible because of segmentation failure, manual measurements will remain the way to evaluate growth.

There is the general principle that identical measurement techniques should be used for baseline and follow-up. This means that, if volumetry fails at a later point in time and manual diameter measurements must be performed, earlier measurements have to be repeated using manual diameter measurements.

### Limits of measurements

There will be situations in which measurements of nodule size are difficult because the margins of a nodule cannot be assessed on non-contrast CT, e.g., in the case of a nodule that causes atelectasis, or because the definition of size is not clear-cut, like in cystic or cavitary lesions. In situations in which measurements are impossible, a multidisciplinary team (MDT) can be used to define management of such lesions, and should follow-up be chosen, visual estimation of change should be the guiding principle.

### Measurement repeatability

Measurement repeatability is determined from two independent measurements, ideally on two LDCT scans taken on the same day. It is usually visualised using Bland-Altman plots that provide the 95% confidence interval (95% CI) for the difference between repeated measurements of the same nodule. This 95% CI is also called the repeatability coefficient (RC), which is related to the standard deviation $\sigma$ of a single measurement as follows:

1 $${RC}=1.96\sqrt{2}\sigma$$

Measurement repeatability for volumetry is dependent on software, complexity of the nodule and to a lesser degree, on scanning technique [7]. Data is available for metastases, which most often present as solid nodules. Data on subsolid or cystic nodules is, to our knowledge, not available.

Older volumetry software had a repeatability coefficient of 20–25%, independent of nodule size [8]. For nodules that were excellently to satisfactorily segmented by the volumetry software, however, the upper limit of the 95% CI was 13.4% [7]. With more modern nodule segmentation software and increased scanner resolution, better repeatability can be expected. For the ESTI nodule recommendation, we conservatively assume a size-independent repeatability coefficient of 15% for well-segmented nodules.

Measurement repeatability for manual diameter measurements performed by the same observer varies between 1.3 mm and 1.7 mm [6]. We therefore assume repeatability of 1.5 mm for manual diameter measurements.

### CIs for volume and diameter measurements

A repeatability coefficient is not identical to the 95% CI. It is larger by $\surd 2$, as can be derived from Eq. (1).

For diameter measurements, the repeatability coefficient is an absolute number and assumed constant at 1.5 mm for this nodule management recommendation. This results in a 95% CI for single manual diameter measurements of ±1.1 mm, independent of nodule size. If three orthogonal manual measurements are averaged, then the 95% CI is reduced by $\surd 3$ and drops to ±0.6 mm.

For volumetry, the repeatability coefficient is a percentage and is assumed to be constant at 15% for this nodule management recommendation. This implies that the absolute repeatability coefficient for volumetry (and therefore the CI) will become volume-dependent. Since volumes can be translated back to effective diameters, the 95% CI for effective nodule diameter will vary with nodule size: ±0.2 mm for a nodule volume of 100 mm^3^ (effective diameter 5.8 mm), ±0.3 mm for nodule volumes of 250 mm^3^ (effective diameter 7.8 mm) and ±0.3 mm for 500 mm^3^ (effective diameter 9.8 mm).

This indicates that effective diameters can be determined much more precisely from volumetry (95% CI ± 0.2–0.3 mm) than average diameters from three orthogonal manual diameter measurements (95% CI ± 0.6 mm). Even if volumetry is not perfect, the repeatability coefficient stays around 25% [7], and the 95% CI for effective diameters will only increase to between ±0.3 and 0.5 mm for the nodule sizes described above. This is still superior to manual measurements and a strong argument to prefer volumetry whenever possible.

### Definition of growth

Growth definitions can be based on absolute change or percentage change in diameter or in volume over a defined time interval (*t*). Since most lung tumours exhibit exponential growth [9], we chose volume-doubling time (VDT) as the preferred measure of growth rate. This approach has also been used in the European position statement on lung cancer screening (EUPS) [10]. The VDT defines the time a tumour takes to double in volume. VDT can be derived from the ratio of volume *V*_0_ at baseline and volume V after a follow-up time interval *t*:

2 $${VDT}=t\cdot {lg}2/{lg}({V/V}_{0})$$

If multiple time points are available, a logistic regression across all time points should be used to more accurately determine the VDT. If that is not possible, the baseline and last measurement should be used to calculate VDT.

Because invasiveness and aggressiveness are mainly related to the solid component of a nodule, we chose to focus on the growth of this solid component to trigger management decisions. Whenever the segmentation of a solid nodule or the solid component of a part-solid or cystic nodule is acceptable at visual inspection, the VDT is used as a measure of growth rate.

If segmentation accuracy is unacceptable, diameters of a solid nodule or the solid component of a part-solid nodule must be manually measured as the largest orthogonal dimensions in axial sections and in the craniocaudal direction. The change in average diameters between two scans defines growth. Growth rate is given as the change in diameter in mm per year. If a follow-up after one year is not yet available, the change in diameter at the longest available time interval is used instead.

### VDT as a surrogate of aggressiveness

Recent data from Korea [11] found that malignant solid nodules grow with a median VDT of 248 days but can exhibit a wide range of growth rates, ranging from as short as 31 days, found for a solid or micropapillary tumour, to longer than 600 days (maximum 2122 days), found in a substantial number of acinar and papillary subtypes.

The same study found that part-solid nodules grew with a median VDT of 665 days and non-solid nodules grew with a median VDT of 648 days. It must be noted, however, that the study used a VDT calculated for the whole nodule, including the ground-glass component, not only for the solid component.

Subsolid nodules are generally less aggressive lesions, for which most guidelines recommend a conservative approach, in line with results demonstrating that long-term surveillance of these nodules is a safe strategy [12, 13].

### Risk of stage shift and overtreatment

A major stage shift in the setting of lung screening occurs if a tumour stage T1a (< 1 cm) at baseline develops into a tumour stage ≥ T1c (≥ 2 cm) during follow-up, or if lymphatic or distant metastases develop.

Larger lesion size increases the risk for lymphatic and distant metastases. Stage shift is also more likely in more aggressive, rapid-growing tumours, after larger follow-up intervals, and with a nodule size that is close to the border between stages. In subsolid nodules, the formation of a solid component indicates invasiveness.

Overtreatment in a lung cancer screening setting happens if a pulmonary malignancy is treated without affecting patient survival. It is most likely to occur in slow-growing tumours with a low propensity for metastases or in patients who suffer from substantial co-morbidities that will influence survival more than the lung tumour itself.

### Measurement errors for growth estimation

**Fig. 3:**
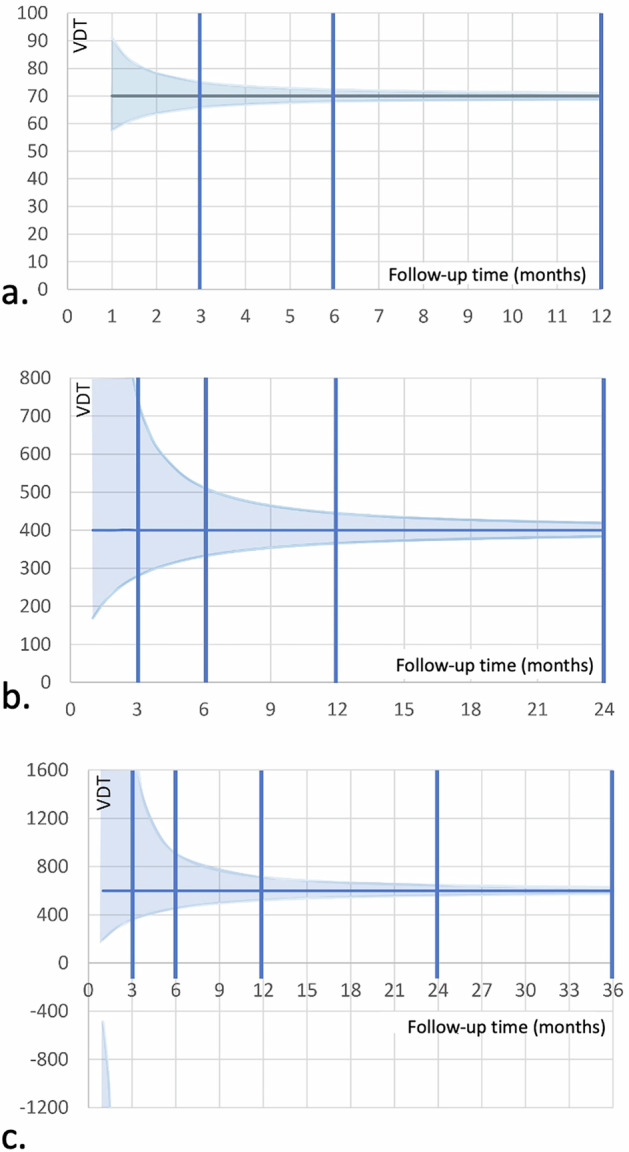
95% CI for VDTs, shown in light blue, based on a repeatability of 15% for volumetric measurements. VDT measurement errors are calculated from two volumetry measurements at baseline and follow-up for true VDTs of 70 d (**a**), 400 d (**b**), and 600 d (**c**). Typical follow-up intervals are indicated by vertical lines. For slow-growing lesions with a long VDT (**c**), the 95% CI extends across infinity for very short follow-up times, indicating that it is then impossible to determine whether the nodule has grown or shrunk. VDTs can be more accurately determined with longer follow-up (**b**, **c**) or if the true VDT is short (**a**)

At least two measurements are necessary to measure growth. Measurement errors at these two points will lead to uncertainties in the determination of growth rates and VDTs. The 95% CI for VDT—a consequence of measurement errors—can be determined from the repeatability coefficient of the two volume measurements *V* and *V*_0_, but will also depend on the true VDT of the lesion and the time interval for follow-up (Fig. 3).

We assume that the measurement error for volumetric size ratios *V*/*V*_0_ can be approximated by a Gaussian distribution around the true value. Because of the logarithmic term in Eq. (1), however, the error margins (95% CIs) for VDT are asymmetrically distributed around the true VDT with wider upper margins and smaller lower margins, especially for short follow-up times (Fig. 3).

The accuracy of VDT estimation increases when longer follow-up intervals are used or when VDT is short. This implies that the VDT of rapidly growing nodules can be estimated more accurately, even with short follow-up intervals (Fig. 3a). Slow-growing nodules require longer follow-up intervals for accurate measurements (Fig. 3b, c). At short follow-up intervals, it may not be possible to determine whether a slow-growing nodule actually grows or shrinks because the 95% CI for the calculated VDT will extend across infinity (Fig. 3c).

If more than one follow-up has been performed, growth curves can be fitted through the various measurements, and resulting errors will decrease with increasing number of follow-up scans.

## Management of solid nodules

### Benign morphology (solid nodules)

In case of clear features of benign disease [14] (central, diffuse or popcorn-like calcification, fat content, typical intrapulmonary lymph node morphology), this nodule will not undergo further evaluation and will thus not affect participant management. If no other lesion is present, regular annual screening is performed.

### Morphological criteria suggestive of malignancy (solid nodules)

Morphological criteria of a nodule may suggest a higher likelihood of being malignant. These criteria include spiculation, architectural distortion (pleural tag, fissure displacement), cystic component, or narrowed vessels with the lesion [14].

While these criteria are known to show a substantial inter-observer variability, at least in subsolid lesions, a higher likelihood for malignancy was consistently found for those cases in which observers indicated suspicious morphology [15]. The presence of morphological criteria suggestive of malignancy will upgrade the nodule to the next higher risk category. For this purpose, an AI algorithm suggesting high risk for malignancy could be used in appropriate settings.

### Management at baseline (solid nodules)

Nodule management is based on risk categories that are related to the risk of major stage shift within 1 year. These risk categories (very low, low, indeterminate, high) are primarily based on size ranges similar to those from other guidelines and on the presence of morphologic criteria suggestive for malignancy that will upgrade a nodule to the next higher risk category.

A nodule in the high-risk category will trigger diagnostic workup and will be seen as a positive screen result. If no morphologic malignancy criteria are present, solid nodules above a size threshold of 500 mm^3^ (or 10 mm average diameter if volumetry fails) will fall into this category. The size threshold was chosen at 500 mm^3^ as a compromise between unnecessary workups for benign lesions and potential stage shift to T1c at follow-up, and lies between the recommendation of Fleischner [16] and LungRADS v2022 [17] and is identical to the original definition in Nederlands–Leuvens Longkanker Screenings Onderzoek (NELSON). The size threshold drops to 250 mm^3^ (or 8 mm average diameter if volumetry fails) if additional morphologic malignancy criteria are present, and then is identical to the recommendations of Fleischner [16] and the EUPS [10].

This approach makes sure that only potential T1a lesions remain in the screening program, while lesions with a potentially higher stage are worked up.

### Intervals for 1st follow-up (solid nodules)

Follow-up intervals for those lesions that are not immediately referred to diagnostic work-up are chosen to find a balance between the risk of a stage shift for a malignant lesion and the number of follow-up scans needed for the whole screening cohort. We chose similar size thresholds and follow-up times to other guidelines [16, 17] if no additional morphologic malignancy criteria are present: regular 1-year follow-up is recommended for nodules < 100 mm^3^ (very low-risk nodule; negative screen result), 6-month follow-up is recommended for lesions ≥ 100 mm^3^ and < 250 mm^3^ (low-risk nodule; indeterminate screen result), and 3-month follow-up is recommended for lesions ≥ 250 mm^3^ and < 500 mm^3^ (intermediate-risk nodule; indeterminate screen result). These thresholds correspond to effective diameters of 5.8 mm, 7.8 mm, and 9.8 mm, which are very close to the size thresholds of 6 mm, 8 mm, and 10 mm given in other guidelines [16, 17] and which are used in this nodule management recommendation if volumetry fails.

To estimate the risk for major stage shift during the chosen follow-up interval, namely from T1a to T1c, we calculated at which growth rate (VDT) this would occur for the various size categories. These worst-case scenarios assume a baseline nodule size just at the size threshold towards the next larger nodule size category.

A malignant nodule with a baseline size close to the threshold of 100 mm^3^ (5.8 mm) would reach stage T1c (2 cm diameter) after regular 1-year follow-up if its VDT were 68 days or lower. A nodule at the threshold of 250 mm^3^ (7.8 mm) would reach stage T1c after 6 months with a VDT of 45 days, and a 500 mm^3^ (9.8 mm) nodule would reach stage T1c after 3 months with a VDT of 30 days. Only lesions that grow faster than the VDT thresholds described above will exhibit a stage shift to ≥ T1c at the first follow-up. None of the adenocarcinomas in the study by Park et al showed a VDT of 30 days or below [11], indicating that a major stage shift in the largest nodule category between 250 mm^3^ and 500 mm^3^ will be an exceptional event. But even in the categories of smaller nodules, the data by Park et al suggests that a major stage shift during follow-up will be found in less than 5% of malignant lesions.

At the same time, the percentage of malignancies among small nodules is very low and decreases substantially with nodule size [2, 18]. The National Lung Screening Trial (NLST) found that cancers could be confirmed among nodules of 4–6 mm in size in only 12 of 3822 nodules (0.3%) at baseline. These numbers increased to 46 of 1959 nodules (2.3%) at baseline for lesion size between 7 mm and 10 mm [18]. This indicates that finding a significant stage shift at first follow-up will be a very rare event with our recommendation.

### Substantial growth that triggers diagnostic work-up (solid nodules)

Substantial growth is defined such that the likelihood of sending benign or slow-growing nodules (VDT ≥ 600 days) to workup is kept to a minimum.

In case that automatic nodule segmentation is successful, substantial growth would ideally be defined by a single growth threshold, a VDT of 600 days [10]. Any nodule that grows faster, with a VDT < 600 days, is sent to diagnostic workup; the rest is kept in follow-up. This ensures that faster-growing nodules are detected while slow-growing nodules remain under further control.

However, measurement variability makes it impossible to accurately determine VDT. As discussed above, VDTs calculated from two volumetric measurements are asymmetrically distributed around the true value (Fig. 3). Measurement variability is higher for short follow-up intervals. This makes it impossible to use a single discriminatory threshold. The VDT thresholds need to be adjusted to the follow-up time, so that the false positive rate, defined as the percentage of cases with a true VDT ≥ 600 days, is kept to a minimum.

**Fig. 4:**
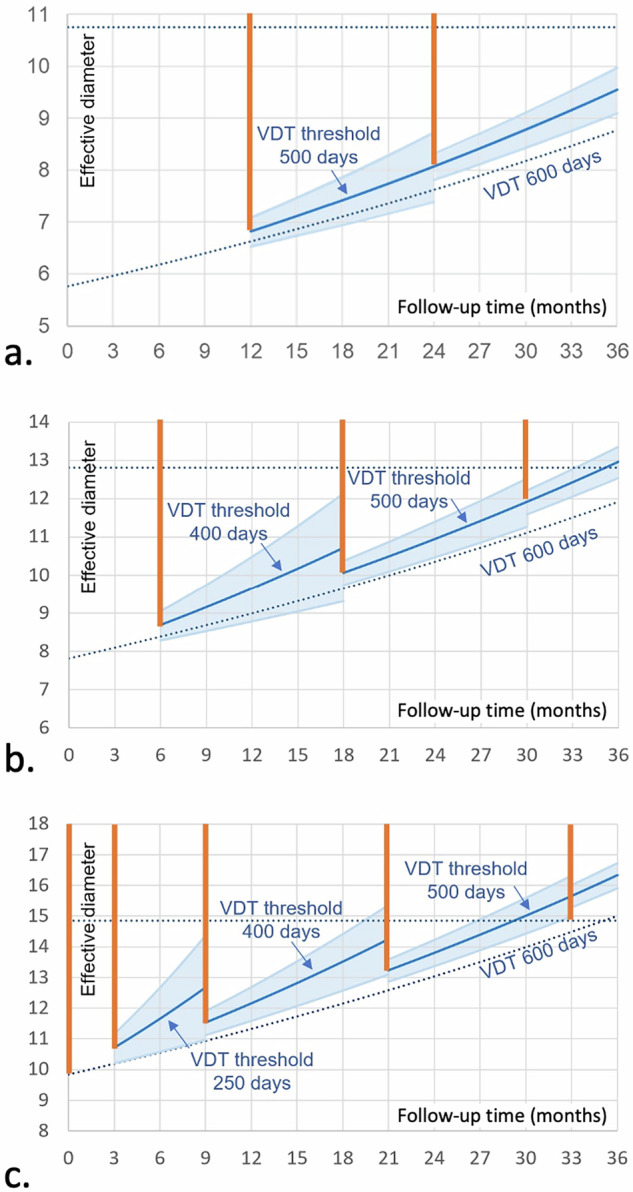
Growth curves for the worst-case scenarios of nodules entering follow-up in each size category: a 99 mm^3^ nodule (effective diameter 5.8 mm) with yearly follow-up (**a**), a 249 mm^3^ nodule (7.8 mm) with a 6-month follow-up (**b**) and a 499 mm^3^ nodule (9.8 mm) with 3-month follow-up (**c**). VDT thresholds at 1st, 2nd, and 3rd follow-up are chosen so that the 95% CI for the calculated VDT (in light blue) remains around 600 days. This ensures that as few benign lesions as possible are sent for workup. The follow-up intervals were adapted so that the interval growth would stay below 5 mm in effective diameter (see dashed horizontal line) for the vast number of lesions during the first two follow-ups. Towards later follow-ups, this 5 mm size threshold is reached even with slow-growing nodules, and triggers referral to an MDT to weigh potential overdiagnosis against cancer risk. The red lines indicate the size ranges that will be sent to workup or MDT at the various follow-up intervals

At shorter follow-up times, the CI for calculated VDTs is wider. Therefore, the VDT threshold is lowered to prevent too many false-positive referrals. We chose a VDT threshold of 250 days for the 3-month follow-up, 400 days for the 6-month follow-up, and 500 days for the regular 12-month follow-up. With these VDT thresholds, the 95% CI limits remain close to 600 days (Fig. 4), given a repeatability of 15% for the volumetric measurements used for calculating VDT.

**Fig. 5:**
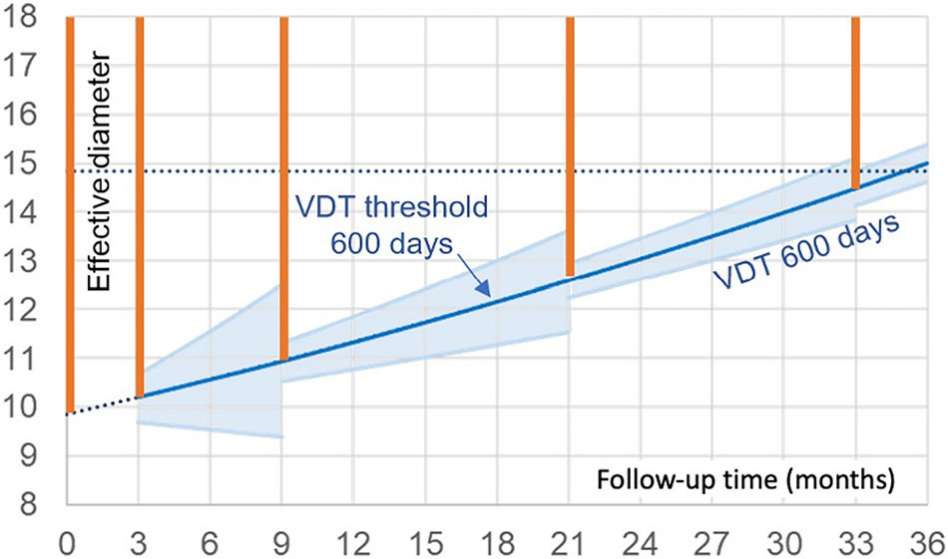
Growth curves for the worst-case scenarios in the indeterminate size category if a constant growth threshold of 600 days is used, like in EUPS [10]. As a consequence of this constant VDT threshold, the lower half of the 95% CI will include nodules that grow slower than the 600-day cutoff. During the 1st, this 95% CI also includes shrinking nodules. This leads to a higher risk of unnecessary workup and overdiagnosis, but will also lead to less growth of the nodules sent to workup. The red lines indicate the size ranges that will be sent to workup

If a constant threshold of 600 days VDT would be used independent of the follow-up interval [10], then substantially more slow-growing and benign nodules will be sent to workup (Fig. 5). This is especially relevant for short follow-up intervals of 3 and 6 months but becomes less pronounced for 12-month follow-up.

**Fig. 6:**
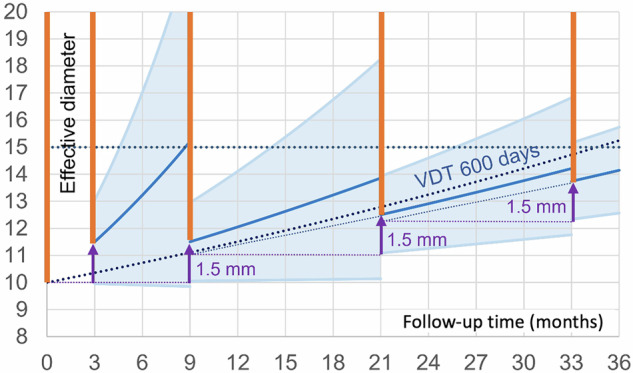
CIs for growth estimates from diameter measurements in those cases in which volumetry is impossible. This situation emulates the LungRADS v2022 management with diameters averaged across two perpendicular measurements. The graph shows the growth curve for the worst-case scenario for a nodule just at the border to the highest risk category. The exponential growth necessary to reach the 1.5 mm growth threshold at the 1st follow-up is indicated by a dark blue line. The worst-case scenario at the 2nd follow-up will occur if the nodule is just below the 1.5 mm growth threshold at the 1st follow-up but grows by 1.5 mm within the following year. The 95% CIs for growth to exceed the 1.5 mm threshold are indicated in light blue. Because the repeatability coefficient for diameter measurements is much less favourable than for volumetry, this 95% CI is very large: the possible true change extends from nearly constant to rapid growth for the 1st and 2nd follow-up, but this span will decrease at later follow-ups. These large CIs imply that a diameter-based management will cause much more unnecessary workup for benign or slow-growing lesions (the portion of the CIs below the 600 days VDT line). At the same time, some malignant lesions will grow to 15 mm despite not having reached the growth threshold at the first follow-up measurement. The red lines indicate the size ranges that will be sent to workup

For cases in which automatic lesion segmentation is unsuccessful, VDT cannot be reliably determined. Substantial growth then implies a visually verifiable increase in average diameter of > 1.5 mm or substantial change in morphology, especially of morphological criteria suggesting malignancy. This diameter growth threshold is similar to that employed by LungRADS v 2022 [17]. Diameter growth thresholds, however, are less sensitive than VDT thresholds and keep more growing lesions in follow-up. Since the 1.5 mm threshold is at the limit of measurement variability, the number of benign lesions sent to workup will increase compared to the VDT approach (Fig. 6). If the change is underestimated in case of a 3-month follow-up, then the malignant nodule will remain in follow-up and may transition to stage T1c, although this is expected to happen in a very small minority of cases (Fig. 6).

### Further follow-up (solid nodules)

Nodules that shrink in size (negative VDT), have a very low risk of stage shift within one year and return to regular 12-month screening intervals.

Nodules that do not show substantial growth as defined above, are down-staged in risk category and are followed at the respective intervals: an intermediate-risk nodule with no substantial growth after 3 months will be categorised as low risk and followed in another 6 months. If substantial growth occurs at this second follow-up (VDT < 400, or growth > 1.5 mm in average diameter relative to baseline if volumetry is deemed inaccurate), diagnostic workup is advised. Otherwise, the nodule is again down-staged to very low risk and is followed at the regular 12-month interval.

The same holds true for a low-risk nodule for which the 6-month follow-up showed no substantial growth. The nodule is then downgraded to very low risk and followed up at 12 months. If substantial growth occurs at this 12-month follow-up (VDT < 500, or yearly growth > 1.5 mm in average diameter in the previous year if volumetry is deemed inaccurate), diagnostic workup is advised, otherwise, the participant remains in regular annual screening.

The VDT calculated for each lesion will be more accurate the more follow-ups have been acquired because VDT can then be calculated using an exponential fit through three or more time points. As a result, the VDT estimates become precise enough so that any nodule can safely be followed with yearly intervals. Since there will still be some residual measurement error, we chose to keep the VDT threshold at 500 days so that only a minimum of slow-growing lesions is sent to workup.

### Slow-growing solid nodules

Malignant lesions can exhibit very slow growth, even for solid tumours [11]. These slow-growing lesions may not become life-limiting in patients with co-morbidities and a limited overall life expectancy. However, if life expectancy is otherwise good, slow-growing lesions may indeed become life-limiting. We therefore chose to add an absolute growth threshold of 5 mm: if a slow-growing lesion increases in (effective or average) diameter by at least 5 mm, this triggers a decision by an MDT to weigh potential overtreatment against the cancer risk. Through this safety threshold, we aim at avoiding stage-shift in patients with a good life expectancy while reducing aggressive management in patients who might not profit from it.

### New solid nodules

While nodules detected at baseline can be any size and any stage, new nodules should ideally be diagnosed in stage T1a to ensure the best possible outcome for the screening participant. Waiting too long with follow-up may lead to stage shift, while aggressive follow-up will lead to a larger number of unnecessary scans. This nodule management recommendation therefore tries to balance early diagnosis against excessive numbers of follow-up exams.

*New* nodules can be truly new or be present in retrospect. Incident nodules are truly new nodules, meaing no abnormality could be seen on the previous CT scan even retrospectively. Prevalent nodules are “new” nodules that either were too small to be called or have not been previously detected but were present in retrospect.

It has to be noted that most new nodules (55% in the NELSON cohort) will resolve [18]. Only 3% of all new nodules at 1-year follow-up were found to be malignant [19], with an absolute cancer rate of only 0.2% (14 cancers in 7295 participants). However, lung cancer was found in 22% of solid nodules ≥ 30 mm^3^ that were visible in retrospect [20].

#### “New” (prevalent) solid nodules

For prevalent nodules, growth is usually evident and requires no volumetric assessment. Such nodules should be sent for further workup. In dubious cases, the VDT criteria mentioned for nodules at 1st follow-up should be used if the lesion at the current and previous scan is suited for volumetry (well-segmented by the volumetry algorithms). The VDT for the appropriate time interval has to be used; if a prevalent nodule is newly detected, for example, on a 6-month follow-up scan, the VDT threshold of 400 days for this 6-month interval has to be used. If such a dubious lesion cannot be evaluated by volumetry, short-term follow-up after 3 months is recommended.

#### New (incident) solid nodules

Incident nodules must have grown from a size below the detection threshold to the size found at follow-up. The shorter the follow-up period, the faster the growth rate must have been (Table 1). The estimates of the VDT required to reach a certain nodule size at detection will strongly depend on the estimated initial nodule size in the previous scan. If no nodule can be seen on the previous scan, even in retrospect, the initial nodule size would have had to be below the detection limit of the CT scanning technique. Table 1 compares the VDTs required to reach the size at detection, calculated for an initial size of 4 mm^3^ (2 mm effective diameter) and 15 mm^3^ (3 mm effective diameter). For most modern scanners, even at low-dose mode, the spatial resolution is below 0.7 mm, and the detection limit is around 4 mm^3^. This detection limit, however, may not be reached in low-dose mode, which is why we also provided the 15 mm^3^ limit used in previous studies [19].

The VDTs have to be extremely fast to reach even a volume of 30 mm^3^ at 3-month follow-up. Larger nodule size, especially for new nodules occurring at short follow-up intervals, suggests very rapid growth, which makes infection or an inflammatory lesion much more likely than malignancy. Even for longer follow-up, most truly new nodules will still be infectious or inflammatory. This is supported by the findings in NELSON: even though a very conservative detection limit of 15 mm^3^ was used [19–21], only 10% of lesions ≥ 500 mm^3^ turned out to be malignant, and in none of the smaller size categories, a malignancy rate of 11% at 1-year follow-up was exceeded. This supports the fact that most new nodules are benign. On the other hand, a substantial number of the malignancies found in new nodules (10/14) in NELSON manifested as nodules ≥ 50 mm^3^ at 1-year follow-up, and all but one new malignancy measured 27 mm^3^ or larger at detection [19]. This implies rapid growth of most of these new nodules.

We therefore chose a size threshold of 30 mm^3^, similar to the one used in nodule management with EUPS [10], to induce accelerated follow-up in these new nodules. As a follow-up interval, we chose 3 months, which implies that even at a very short VDT of 50 days, the effective nodule diameter will grow by approximately 50%. Nodules that do not show substantial growth (VDT < 250 days or visually verifiable growth > 1.5 mm or change in morphology) will return to 12-month follow-up. Nodules < 30 mm^3^ will undergo regular yearly follow-up. Only very fast-growing nodules with a VDT ≤ 51 days will start from 30 mm^3^ and exceed the size threshold to stage T1c after 1 year of follow-up. This ensures that the risk of stage shift in new small nodules is minimised with our protocol, while the number of unnecessary scans remains minimal.

## Management of subsolid nodules

Subsolid and cystic nodules are generally less aggressive lesions and may be managed with a conservative approach with long-term surveillance [12, 13].

### Benign morphology (subsolid nodules)

Pulmonary findings suggesting an infectious or inflammatory process may be so specific that no further evaluation is necessary, and participant management will not be affected. This holds true for tree-in-bud nodules or ground glass in a distribution suggestive of interstitial lung disease. Other findings suggesting an infectious or inflammatory process will undergo a short-term follow-up at 1 month to distinguish between persistence or regression. Such findings include segmental or lobar consolidations, infiltrates, or multiple part-solid nodules without other morphologic criteria suggestive of malignancy.

### Morphological criteria suggestive of malignancy (subsolid nodules)

Like with solid nodules, morphologic criteria may suggest a higher likelihood for malignancy [14]. These criteria used in this recommendation include: spiculation, architectural distortion (pleural tag, fissure displacement), bronchial cut-off, cystic component, bubble-like lucencies, narrowed vessels within the lesion, and ground glass component of ≥ 3 cm. The presence of suspicious morphology will upgrade the nodule to the next higher risk category. We added the presence of a ground glass component of ≥ 3 cm in order not to miss a growing diffuse lepidic tumour and allow for a 6-month follow-up, even if no solid component is present. Despite high inter-observer variability for the presence of these morphologic criteria, suspicious morphology consistently indicates a higher malignancy risk [15].

### Management at baseline (subsolid nodules)

Nodule management at baseline is based on the size of any solid component and is further modeled after that for solid nodules. This was done because solid components in subsolid nodules often indicate invasive cancer, while overall growth remains slow [12, 13]. If this solid component can be segmented well and is measurable by volumetry, the same criteria and follow-up intervals are used as those for solid nodules. If this solid component is not measurable by volumetry, manual diameter measurements are necessary.

Manual diameter measurements, however, imply substantially higher measurement variability [6]. For this reason, growth is defined as a visually verifiable increase in size of an existing solid portion of ≥ 1.5 mm or a substantial change in morphology. If in doubt, follow-up is advised, given that the true VDTs of such lesions are generally longer than those of solid lesions. Manual measurements of the solid component of a part-solid lesion should be done in three orthogonal planes and averaged.

Part solid lesions with a solid component ≥ 10 mm have a high likelihood for being infectious but also might be tumours with an increased risk for lymphatic or endobronchial spread. In these cases, a 1-month follow-up is advised with workup if the lesion grows or stays constant.

### New subsolid nodules

New (incident) non-solid nodules (ground glass) are usually infectious. Even if (pre-)malignant, they grow very slowly. For this reason, non-solid lesions will undergo no special follow-up, which implies a next scan after 12 months. For new part-solid nodules, the same rules apply as those for new solid nodules, but based on the size of the solid component and not the whole nodule. This implies that any truly new part-solid nodule with a solid component ≥ 30 mm^3^, or ≥ 4 mm in diameter if volumetry is not feasible, will be sent to a 3-month follow-up. If further growth occurs, workup is advised, otherwise, nodules return to a 12-month follow-up.

## Management of cystic nodules

Thin-walled, unilocular cysts with a wall thickness of < 2 mm are considered benign.

All other cysts are morphologically suspicious. Management of cystic lesions follows that of solid or subsolid lesions, depending on the characteristics of the nodular component. The presence of a cystic component, however, is counted as a morphologic feature suggestive of malignancy, which leads to upgrading the cystic nodule to the next higher risk category. This implies that any suspicious nodule with a cystic component will be followed at a 6-month or shorter interval. A one-month follow-up in part-solid lesions with cystic components is only recommended if pneumonia is a realistic differential diagnosis.

## Management of airway nodules

Mucous plugging affecting multiple segments or air bubbles within endoluminal airway abnormalities are indicators of benign disease. Mucus in the trachea and proximal airways often presents as a drop-like configuration in a (semi-)sagittal plane adapted to the longitudinal direction of the airway.

Endoluminal lesions in the segmental or smaller bronchi are usually mucous plugs and are managed like a small solid nodule of the maximum diameter of the affected bronchus. Most of such lesions will be assigned to the lowest risk group (negative screening result) and receive the regular 1-year follow-up. Lesions in a bronchus larger than 6 mm will be placed in the low-risk category and followed after 6 months. Major stage shift is unlikely during follow-up, given their small size.

Focal endotracheal and proximal endobronchial abnormalities are classified as intermediate risk and followed up after 3 months. Many will still be mucous or a benign polyp, but this management makes it possible to detect fast-growing tumours that may otherwise become inoperable.

Because of the difficulty to consistently measure bronchial lesions, change in diameter or calculation of VDT is no viable option. Persistence or growth at follow-up will result in referral to an MDT. Regression will place the nodule in the lowest risk category (negative screening result).

Truly new airway nodules will be followed like airway nodules at baseline.

## Conclusion

This nodule management recommendation is designed to reduce unnecessary workup for benign or slow-growing nodules while keeping the risk of stage shift to ≥ T1c low. It is based on volumetry and VDT calculations for those nodules in which volumetry is feasible. VDT thresholds at follow-up are dependent on follow-up interval to accommodate potential measurement errors. This should ensure excellent survival chances for those individuals diagnosed with lung cancer at follow-up but reduce the number of individuals that receive unnecessary workup or treatment.

## Abbreviations

CT: Computed tomography
ESTI: European Society of Thoracic Imaging
EUPS: European Position Statement on lung cancer screening
LDCT: Low-dose CT
MDT: Multidisciplinary team
NELSON: Nederlands–Leuvens Longkanker Screenings Onderzoek
VDT: Volume-doubling time
